# Macrophage Derived Platelet Activating Factor Implicated in the Resolution Phase of Gouty Inflammation

**DOI:** 10.1155/2014/526496

**Published:** 2014-09-24

**Authors:** Darshna Yagnik

**Affiliations:** Department of Natural Sciences, School of Science and Technology, Middlesex University, The Burroughs, London NW4 4BT, UK

## Abstract

Human blood derived *in vitro* differentiated monocytes or macrophages are a population of cells which have been investigated over the years to determine the role these cells play in the resolution phase of gout. Macrophages are able to phagocytose monosodium urate monohydrate (MSU) crystals without releasing inflammatory factors. This study analysed macrophage platelet activating factor secretion and its possible role in the pathway of gout resolution. Analysis of sunatants from *in vitro* differentiated macrophages stimulated with MSU crystals revealed the secretion of platelet activating factor (PAF)  1.54 ± 0.10 mean ± SEM; ng/mL per 10^6^ cells. This secretion was absent in immature monocytes treated similarly. When these monocytes were pretreated with recombinant human PAF-acetylhydrolase (rhuPAF-AH) and MSU crystals resulted in TNF*α* suppression. Addition of WEB2086, a platelet activating factor (PAF) antagonist, to differentiated macrophages with MSU crystals unmasked TNF*α* secretion 0.7 ± 0.06 mean ± SEM; ng/mL per 10^6^ cells. This study identifies a role for PAF and the PAF receptor antagonist in the pathway by which macrophages ingest MSU crystals and resolve the concomitant inflammation.

## 1. Introduction

An acute attack of gouty arthritis includes clinical features such as joint inflammation, erythema, fever, and extreme, unbearable pain resulting in reduced mobility of the afflicted joint [1]. Prophylactic therapy is recommended to control gouty arthritis flares [2, 3]. The symptoms are caused by monosodium urate monohydrate (MSU) crystal deposition into the intra-articular joint spaces. Crystal precipitation provokes mass polymorphonuclear (PMN) leukocyte infiltration into the joints where these cells release inflammatory cytokines which mediate joint damage [4, 5]. However, in the absence of clinical intervention, gout is self-limiting in nature and is able to spontaneously resolve over a period of a week [6–9]. The exact theory behind the resolution phase of gout remains an enigma although macrophages and their cofactors have been implicated in the mechanism [10–12].

Platelet activating factor (PAF, 1-0-alkyl-2-acetyl-sn-glycero-3-phosphocholine) was discovered in 1972, it is known as one of the most potent phospholipids, involved in platelet aggregation and anaphylaxis [13]. It is produced by a variety of immune cells including monocytes/macrophages, neutrophils, eosinophils, basophils, and platelets [14, 15]. There are two main pathways which lead to cellular synthesis of PAF, the de nova pathway and remodelling pathway. Inflammatory or immune stimuli induce PAF primarily via the remodelling pathway whereby membrane bound PAF-acetylhydrolase (PAF-AH) which is an extracellular phospholipase A2 catalyses the transfer of an acetyl residue from acetyl-CoA to 1-alkyl-sn-glycero-3-phosphocholine (lyso-PAF). This is produced by the action of phospholipase A2 on phosphatidylcholine [16–18].

However, the full mechanisms and the exact role of various enzymes on PAF degradation have not been fully elucidated to date.

To exert its effect, PAF binds directly to a specific G protein coupled receptor and is an important mediator of juxtacrine and paracrine signals between cells [19]. PAF is well regarded as a potent mediator of inflammatory pathologies such as acute pancreatitis [20], diabetes [21], and renal failure [22]. However, recent studies suggest PAF could also play a crucial role in regulating immune responses. Studies have highlighted the necessity of PAF receptor activation in deactivation of ultraviolet B (UVB) induced suppression of hypersensitivity responses [23]. In addition, PAF administration to equine alveolar macrophages increased phagocytic capacity and superoxide anion production [24]. Moreover, during immunological defence, PAF can play an immunomodulatory role including monocyte or macrophage degranulation, cytokine release, phagocytosis, and cell adhesion [25, 26].

## 2. Materials and Methods

### 2.1. Reagents and Antibodies

WEB2086 was purchased from Tocris Bioscience (UK). Human TNF*α* ELISA kit was purchased from R and D systems Europe. PAF ELISA kit was purchased from Blue Gene Biotech, China. LPS, antimonoclonal anti-human CD62, anti-mouse IgG FITC, and mouse IgG1 were purchased from Sigma-Aldrich (Poole, UK). MSU crystals were purchased from Enzo Life Sciences, Germany. HUVEC cells were purchased from ATCC cell lines, UK. Recombinant human PAF-acetylhydrolase was purchased from Sigma-Aldrich, USA.

### 2.2. Monocyte Isolation Procedure

Leucocyte rich cones were obtained from volunteer donors collected from NHS Cord Blood and Transplant Bank at Colindale, London. Briefly, the cones were washed with PBS to harvest leucocytes. The leucocyte rich cells were then spun on Histopaque at 1200 RPM for 20 mins. The mononuclear portion was obtained and washed in HANKS balanced solution. Cells were counted and cultured into 24 wells at 4 × 10^6^/mL. Cells were allowed to adhere for an hour after which nonadherent cells were washed and full media replenished with Dulbecco's media containing AB serum. Differentiated macrophages were determined by light microscopy and flow cytometry phenotypic analysis of differentiation markers. Cells were washed and fed at days 1, 3, 5, and 7 of culture and stimulated with LPS (10 *μ*g/mL) or MSU crystals (0.5 mg/mL) with or without pretreatment with WEB2086 for 16–24 hours after which supernatants were collected and stored in aliquots at −70°C prior to analysis. Pretreatment with rhuPAF-AH at concentrations of 2.5–20 *μ*g/mL was carried out for half an hour prior to addition of MSU crystals to monocyte cells. Supernatants were collected and analysed for TNF*α* after 16 hours.

### 2.3. Enzyme Linked Immunosorbant Assays (ELISA)

PAF and TNF*α* levels in cultured supernatants were determined by ELISA (purchased from Blue Gene Biotech, China, and R and D Systems Europe, resp.) using manufacturer's recommendations. For PAF determination, culture supernatants were pretreated with balance solution provided in the ELISA kit, mixed, and then processed according to manufacturer's instructions. The antibodies to PAF formed solid phase ELISA. All samples were measured in duplicate, with results expressed as the mean ± SEM cytokine concentration (ng/mL) from at least 4 experiments.

### 2.4. Flow Cytometric Analysis

Indirect flow cytometric analysis was performed as previously described (12), using primary antibodies at 10 *μ*g/mL and secondary antibodies at 1 : 64 dilution. Endothelial cells were detached by trypsin/EDTA and the trypsin activity was quenched in excess growth medium supplemented with serum. Monocyte/macrophage cultures were detached by 15-minute incubation in ice-cold phosphate buffered saline (PBS) containing 2.5 mM of EDTA, followed by scraping and washing in growth medium containing serum. Data analysis was performed using a flow cytometer analyser purchased from Beckton Dickinson (BD), UK. Results were analysed using BD Cell Quest Pro software.

HUVEC E-Selectin expression was calculated by dividing the staining intensity (mean fluorescent intensity (MFI)) of E-Selectin by the staining intensity of class-matched control antibody, thus resulting in a relative fluorescent intensity (RFI) of expression (where an RFI of 1.00 is equivalent to no antigen expression). Results were expressed as the mean ± SD RFI from at least 3 separate experiments.

### 2.5. Statistical Analysis

Analysis of inhibition by WEB2086 on TNF*α* secretion and endothelial cell E-Selectin expression was carried out using 1-way analysis of variance (ANOVA) with Dunnett's correction.

## 3. Results and Discussion

**Figure 1:**
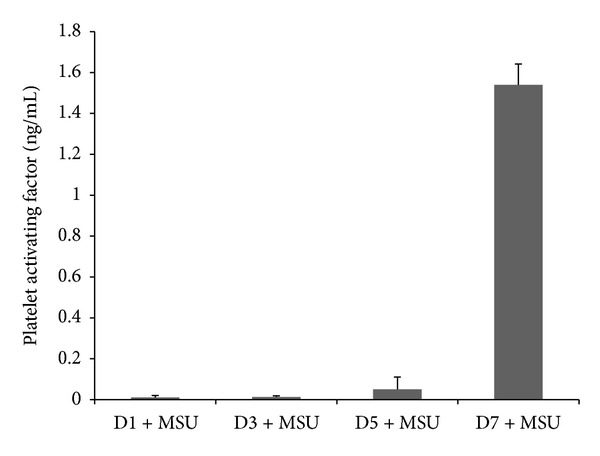
Monocyte differentiation and time course of platelet activating factor (PAF) production after challenge with MSU crystals. Isolated monocytes were differentiated over 7 days* in vitro *and incubated with MSU crystals for 17 hours at day 1 (D1), day 3 (D3), day 5 (D5), and day 7 (D7) of culture. Collected supernatants were analysed for PAF secretion by ELISA. Results are mean and SEM of 4 experiments.

**Figure 2:**
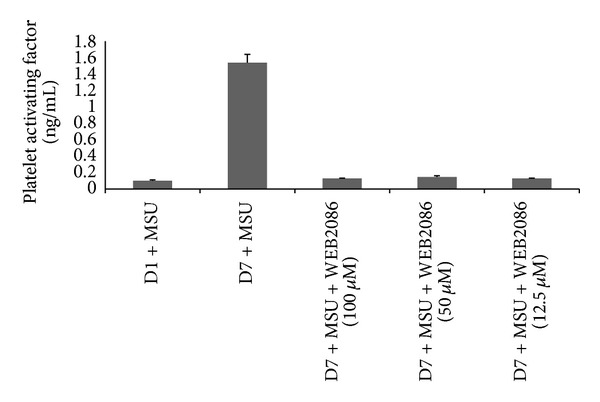
Platelet activating factor (PAF) production by D7* in vitro* differentiated macrophages incubated with MSU crystals and with or without WEB2086 (WEB). Monocytes were cultured for 1 and 7 days and stimulated with MSU crystals and collected supernatants were analysed for PAF by ELISA. Results are mean and SEM of 4 experiments. D7 = supernatants from day 7 macrophages, D1 = supernatants from day 1 Monocytes, and WEB = WEB2086. Macrophages also secreted PAF when incubated with LPS (1.48 ± 0.10 mean ± SEM; ng/mL per 10^6^ cells), data not shown.

**Figure 3:**
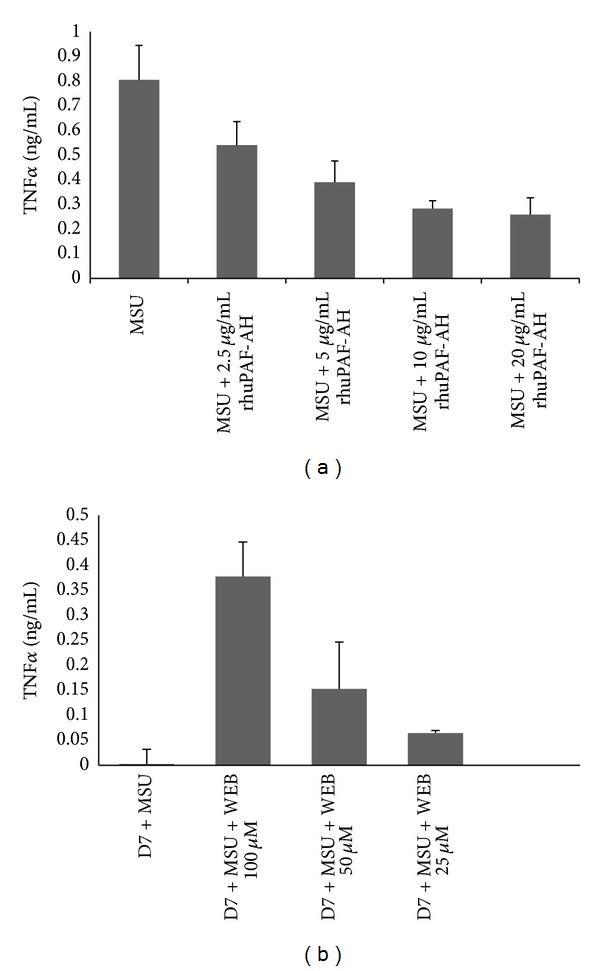
(a) Effect of recombinant human platelet activating factor acetylhydrolase (rhuPAF-AH) on TNF*α* release from monocytes. Freshly isolated monocytes were cultured overnight and treated with rhuPAF-AH at concentrations of 2.5, 5, 10, and 20 *μ*g/mL, respectively, for 45 minutes prior to addition of MSU crystals for 24 hours. Afterwards, collected supernatants were analysed for TNF*α* by ELISA. Results are mean and SEM of 3 experiments. (b). TNF*α* production unmasked by costimulation of* in vitro *differentiated macrophages with MSU crystals and WEB2086. Monocytes were cultured* in vitro* for 7 days and stimulated with MSU crystals for 24 hours with or without the PAF antagonist (WEB2086). At 100 *μ*M, coincubation with WEB2086 and urate crystals caused the release of TNF*α* with *P* = 0.002 (Student's paired *t*-test). Conditioned supernatants were analysed for TNF*α* production by ELISA. Differentiated macrophages were also coincubated with WEB2086 at 100 *μ*M which reduced the amount of TNF*α* unmasked. Values are mean and SEM of 4 experiments. D7 = supernatants from day 7 macrophages, D1 = supernatants from day 1 monocytes, and WEB = WEB2086 (PAF antagonist).

Figure 1 shows that monocytes secrete detectable PAF when incubated with MSU crystals. This detection of PAF occurs at day 5 and peaks at day 7 of monocyte differentiation. The phenotype of monocytes as they differentiate into macrophages has been well characterised and it has previously been postulated that they can acquire an anti-inflammatory phenotype. A similar model showed that at days 5–7 of monocyte culture, upon stimulation with MSU crystals, an anti-inflammatory phenotypic response was enabled [11]. This was corroborated by the detection of TGF*β*1 secretion by human* in vitro* differentiated macrophages in response to MSU crystal challenge. Coincubation of day 7* in vitro *differentiated macrophages with the PAF receptor antagonist (WEB2086) and MSU crystals resulted in a decrease in PAF secretion (Figure 2). To investigate whether PAF production was autocrine suppressive, MSU crystal stimulated macrophages were incubated in the presence of increasing concentrations of the PAF receptor antagonist and this time TNF*α* production was analysed. Inclusion of WEB2086 at concentrations of 25–100 *μ*M in these cultures resulted in peak TNF*α* production (Figure 3(b)). In fact, this TNF*α* production was comparable to that released by day 1 monocytes after MSU crystal stimulation which had been determined in a previous study [11].

Prior investigations have reported that macrophage cell lines and human* in vitro* differentiated macrophages can ingest MSU crystals without releasing inflammatory cytokines (TNF*α*, IL-1*β*, and IL-6) [11, 12]; indeed, it was identified that instead macrophages produced anti-inflammatory TGF*β*1. In addition, it was observed that TGF*β*1 was able to inhibit endothelial cell activation and E-Selectin expression [12]. Essentially in this study coincubation of endothelial cells with anti-TGF*β*1 and a mixture of day 1 and day 7 MSU crystal stimulated supernatants unmasked only a partial degree of endothelial E-Selectin reexpression even with using maximum concentrations of neutralising antibody against TGF*β*1. This suggested that other anti-inflammatory factors could also be released by macrophages upon phagocytosis of MSU crystals. Figures 1 and 2 identify macrophage derived PAF in this mechanism. Another anti-inflammatory model whereby macrophages play an active role in regulating inflammation and resulting in PAF mediated activity by macrophages is the phagocytosis of apoptotic neutrophils. Indeed, this mechanism also results in a lack of proinflammatory cytokine release and associated inflammation [27–29]. Pretreatment of freshly isolated monocytes with rhuPAF-AH and MSU crystals resulted in partial suppression of TNF*α* release. Full suppression of TNF*α* was not expected as manufacturers reference data indicated that rhuPAF-AH cleaved the PAF analogue by approximately 50% at 10 *μ*g/mL, which was observed in Figure 3(a).

It is likely that PAF exerts an immunomodulatory effect on MSU crystal stimulated macrophages as coincubation in the presence of WEB2086 unmasked TNF*α* production, implicating that PAF may be involved in autocrine and paracrine suppression of macrophages.

**Figure 4:**
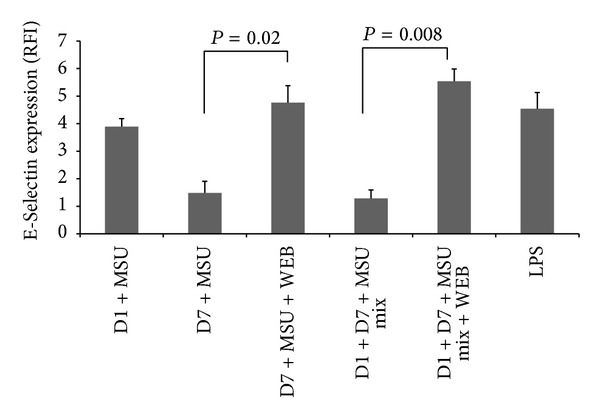
Inhibition of endothelial cell activation by platelet activating factor derived from supernatants from MSU crystal stimulated macrophages. Monocytes or macrophages were cultured for 1 and 7 days, respectively, and stimulated with MSU crystals. Collected supernatants were cocultured with human umbilical vein endothelial cells (HUVEC) for 4 hours at a 1 : 2 dilution or 1 : 1 mix, either alone or in the presence of WEB2086 (a PAF receptor antagonist). HUVEC expression of E-Selectin was measured by flow cytometry. WEB2086 was added to supernatant mixtures in culture at 100 *μ*M which relieved the suppression of E-Selectin seen in the 1 : 1 mix condition (*P* = 0.008 by Student's paired *t*-test). Relief of E-Selectin suppression by supernatants from day 7 macrophages stimulated with MSU crystals was also observed (*P* = 0.02 by Student's paired *t*-test). Results are the mean and SD of 3 experiments. RFI = relative fluorescence intensity. D7 = supernatants from day 7 macrophages, D1 = supernatants from day 1 monocytes, and WEB = WEB2086.

There are studies that highlight PAF's role in endocrine, autocrine, and intercrine signalling [28]. Endothelial activation precedes the inflammatory response by MSU crystals which has been observed* in vitro* and* in vivo* [27]. An antagonist of PAF was able to reduce E-Selectin mediated endothelial activation (Figure 4). Although PAF is well regarded as a potent mediator of inflammation, emerging research suggests that PAF can mediate inflammation control [30–32]. Impaired anaphylactic responses were reported in mice lacking the PAF receptor [23, 26]. Another study showed that mice stimulated with LPS were protected from endotoxin shock when injected with PAF systemically. In these mice, a reduction in proinflammatory cytokine secretion, namely, interferon gamma, IL-1, and TNF*α*, was also observed [32]. Researchers have highlighted the role of PAF mediated interference in microbial associated damage as well. PAF was pivotal in the extent of pathological consequences due to* H. pylori* infection and gastric mucin synthesis [33]. Another report implicated PAF in* Porphyromonas gingivalis*, LPS mediated interference with salivary mucin [34]. Moreover, prophylactic administration of PAF antagonist to rats with gastric injury exerted an anti-inflammatory effect reducing mucosal apoptosis, TNF*α*, and nitric oxide synthase 2 activity and resulted in rapid gastric ulcer healing [35].

In fact, PAF has been identified as a molecular sensor recognising cellular damage and exerting effects on cytokines and COX-2 transcription which resulted in systemic immune suppression [36]. Deposition of MSU crystals into intra-articular joints causes joint swelling and joint damage as well as oxidative stress [2, 3, 5, 37]. Hence, it is feasible that a similar sensory mechanism of cell damage detection by PAF and PAF receptor signalling could be underpinning gouty arthritis.

## 4. Conclusion

This study identifies macrophage derived PAF and PAF related molecules in the noninflammatory phagocytosis of MSU crystals by human blood derived differentiated macrophages. In this model, the PAF receptor antagonist was able to have a number of effects; it was able to inhibit endothelial cell activation measured by E-Selectin upregulation and was also implicated in downregulating TNF*α* secretion by MSU crystal stimulated monocyte cells. Essentially, the immune response to MSU crystals is clearly multifaceted in nature involving a complex interplay by inflammatory and noninflammatory mechanisms involving various leukocyte derived factors which act in coordination during the resolution phase of gouty arthritis. Noticeably, little is known about the role that PAF-acetylhydrolases play in PAF activation and degradation or how PAF exerts its effects through its receptors. These pathways remain to be defined. The model of monocyte, macrophage, and MSU crystal induced inflammation could be used to further examine these molecular mechanisms and unravel the associated PAF signalling and receptor pathways. In conclusion, this study suggests a role for PAF and related molecules in the anti-inflammatory pathway by which macrophages phagocytose MSU crystals.
